# Resistant Maltodextrin Consumption in a Double-Blind, Randomized, Crossover Clinical Trial Induces Specific Changes in Potentially Beneficial Gut Bacteria

**DOI:** 10.3390/nu14112192

**Published:** 2022-05-25

**Authors:** Volker Mai, Alyssa M. Burns, Rebecca J. Solch, Jennifer C. Dennis-Wall, Maria Ukhanova, Bobbi Langkamp-Henken

**Affiliations:** 1 Department of Epidemiology, Emerging Pathogens Institute, University of Florida, Gainesville, FL 32610, USA; jdenniswall@gmail.com (J.C.D.-W.); mukhanova@peds.ufl.edu (M.U.); henken@ufl.edu (B.L.-H.); 2 Food Science and Human Nutrition Department, University of Florida, 572 Newell Drive, Gainesville, FL 32611, USA; aburns22@ufl.edu (A.M.B.); rsolch@tulane.edu (R.J.S.)

**Keywords:** resistant maltodextrin, bifidobacteria, healthy adults, gastrointestinal function

## Abstract

Background: We have previously reported that the addition of resistant maltodextrin (RMD), a fermentable functional fiber, to the diet increases fecal weight as well as the amount of fecal bifidobacteria. Here, we report on the targeted analysis of changes in potentially beneficial gut bacteria associated with the intervention. Objective: The primary objective of this study was to determine the effect of adding 0, 15 and 25 g RMD to the diets of healthy free-living adults on potentially beneficial gut bacteria. Methods: We expanded on our previously reported microbiota analysis in a double-blind, placebo-controlled feeding study (NCT02733263) by performing additional qPCR analyses targeting fecal lactic acid bacteria (LAB), *Akkermansia muciniphila*, *Faecalibacterium prausnitzii* and *Fusicatenibacter saccharivorans* in samples from 49 participants. Results: RMD resulted in an approximately two-fold increase in fecal *Fusicatenibacter saccharivorans* (*p* = 0.024 for 15 g/day RMD and *p* = 0.017 for 25 g/day RMD). For *Akkermansia muciniphila* and *Faecalibacterium prausnitzii,* we obtained borderline evidence that showed increased amounts in participants that had low baseline levels of these bacteria (*p* < 0.1 for 25 g/day RMD). We did not detect any effects of RMD on LAB. Conclusions: RMD supplementation in healthy individuals increases *Fusicatenibacter saccharivorans.* Albeit to a lesser extent, RMD at the higher intake level may also increase *Akkermansia muciniphila* and *Faecalibacterium prausnitzii* in individuals with low baseline levels of those two species. Potential benefits associated with these microbiota changes remain to be established in studies with quantifiable health-related endpoints.

## 1. Introduction

Health benefits of consuming dietary fiber (DF) have been well established and include a decreased risk of obesity, cardiovascular disease, type 2 diabetes and some cancers, especially those of the gastrointestinal (GI) tract. However, questions remain about the generalizability across various DF in mechanisms mediating these health benefits. Some of the previously investigated mechanisms include laxation through fecal bulking, binding and excretion of toxins and support of a balanced gut microbiota with beneficial metabolic capabilities. A better understanding of the contributions of these mechanisms to mediate DF-associated health benefits will facilitate a more personalized approach for adding specific DF that can target health benefits in susceptible individuals. Functional fibers, purified DF that can be added to food or drinks, offer an effective means for increasing DF consumption to reach recommended intake guidelines and confer health benefits. While a wide array of distinct functional fibers have been developed, each functional fiber has distinct characteristics and thus requires data to support its unique health benefits, specific mechanisms involved and acceptability and tolerability by the consumer. Of particular interest, changes in the microbiota during aging might contribute to reduced immune surveillance as well as compromised gut barrier function that can result in increased levels of chronic inflammation. Thus, functional fiber, which targets specific gut microbes might be able to improve symptoms in individuals with chronic inflammatory diseases of the GI tract, including ulcerative colitis (UC).

Resistant maltodextrin (RMD), a water-soluble, fermentable functional fiber derived from heat treatment of cornstarch has been shown to be well tolerated [1]. We have previously shown that supplementing 25 g RMD/day increased DF intake, stool wet weight (WW) and fecal bifidobacteria counts in adults with DF intake below recommendations [2]. To expand upon previous microbiota findings from this intervention study, we now report additional observations of changes in potentially beneficial gut bacteria, particularly, lactic acid bacteria (LAB), *Fusicatenibacter saccharivorans (F. saccharivorans*), *Faecalibacterium prausnitzii* (*F. prausnitzii*) and *Akkermansia muciniphila* (*A. muciniphila*) associated with RMD intake. We selected these taxa due to the recognized or emerging signature in disease states and their potential beneficial effect on the host, as reported in the literature [3,4,5,6,7,8,9]. In these additional analyses, we also stratified participants by pre-intervention baseline levels of the targeted bacteria. We hypothesized that RMD increases levels of targeted bacteria, especially in individuals with lower levels at baseline. The rationale for this approach is that individuals with low or undetectable levels of a potentially beneficial microbial taxon might be more likely to benefit from an effort to increase those numbers than those that already have high levels present.

## 2. Methods

Details of the study have been published previously [2]. In brief, 49 volunteers between the ages of 18 to 50 years old from the southeastern United States completed a randomized, placebo-controlled, double-blind, three-intervention (0 g, 15 g or 25 g) crossover study with a 2-week baseline before each 3-week intervention. This study was conducted from August 2016 to November 2016, was registered at Clinicaltrials.gov #NCT02733263 and conducted according to the ethical guidelines in the Declaration of Helsinki and approved by the University of Florida Institutional Review Board (IRB201501168). Written informed consent was obtained from all participants by trained study coordinators prior to beginning the study. Participants were instructed to mix powdered study supplement in water or a beverage of choice and consume it throughout the day of each intervention. A 2-day total stool collection was obtained during the second week of each baseline and during the last week of each of the three interventions.

### 2.1. Microbiota Analyses

For 16S rDNA-based microbiota analysis, the first stool sample from each day was combined into a single sample per 2-day collection. Total DNA was extracted from 0.1–0.2 g of feces using a modified protocol (QIAamp DNA Mini Kits, Qiagen) that included a bead-beating step [10].

### 2.2. qPCR to Quantify Counts of Targeted Bacteria

Reactions containing 10 ng of DNA and 0.2 μmol/L of each primer [11,12] were carried out in duplicate with an initial melting step at 95 °C for 10 min followed by 40 cycles of 95 °C for 30 s, 58 °C for 60 s and then 72 °C for 60 s.

### 2.3. Statistical Analyses

All analyses were performed on log_10_ transformed counts of targeted bacteria per 10 ng of DNA. For intervention effects on fecal bacteria counts, the difference between the log_10_ counts at the beginning of the first intervention period (baseline) and log_10_ counts at the end of the third week of each intervention was calculated for each participant (Table 1). Comparisons of log_10_ counts at the end of each period among RMD dosage levels (0 g, 15 g or 25 g) were also performed. In exploratory analyses, we stratified participants by baseline levels of each taxon into “low”, “medium” and “high” visually based on the distribution of counts for each targeted probe and compared the effects of RMD in the “low” group by comparing differences of log_10_ counts at baseline with those at the end of the intervention period for RMD dosage levels (15 g or 25 g).

## 3. Results

Using samples collected in a previously reported intervention trial, we successfully quantified RMD-associated changes in three of the four taxa targeted in this study, all of which are commonly found in the human gut microbiota. While the targeted taxa were detected above the minimal detection level in more than 80% of all samples, for some of the targets we failed to detect their presence in one or more samples from a few individuals. While this had minor effects on statistical power to detect potential effects of RMD for some of the targets, overall, these effects were minor as we detected a signal in a vast majority of samples for all four targets.

In the primary analysis, compared to both the baseline and the placebo (i.e., 0 g RMD) period, the log_10_ counts of *F. saccharivorans* increased during the intervention period by more than two-fold upon RMD consumption at either intake level (*p* < 0.05) (Table 1). We did not detect an effect of RMD on any of the other three microbiota targets in the primary analysis.

**Figure 1:**
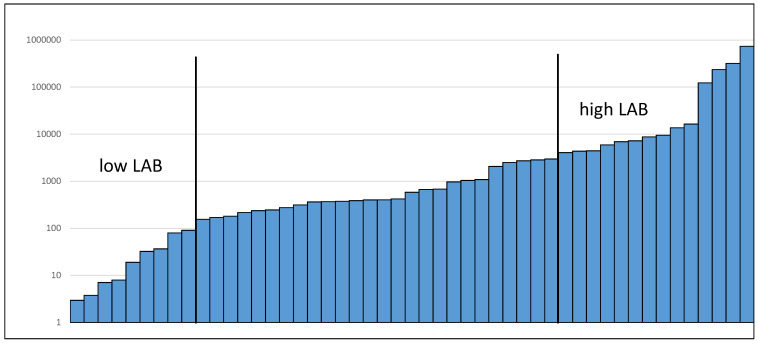
Distribution of LAB at baseline.

**Figure 2:**
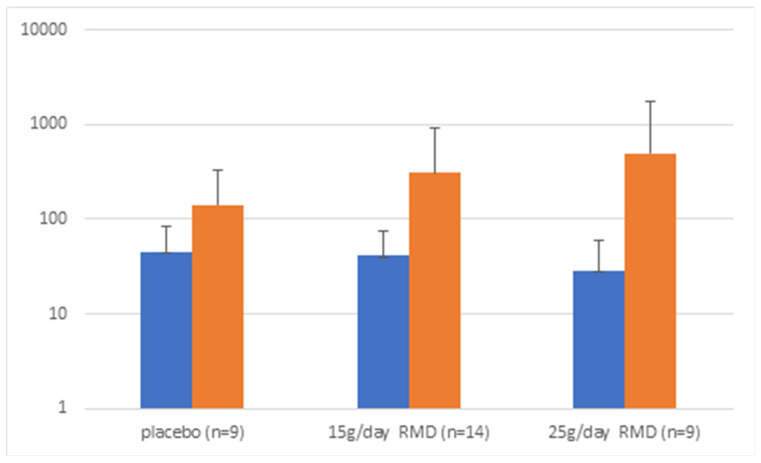
LAB genome copies/10 ng DNA in samples with low LAB content at beginning of each period. Blue: baseline of intervention period; Orange: end of intervention period.

To further explore our data, we then stratified participants by baseline levels of targeted taxa. An example of the stratification into “high” and “low” baseline levels based on the distribution is shown for LAB (see Figure 1). While for LAB there appeared to be more of an increase during the two RMD periods compared to the placebo and this difference was not statistically significant (Figure 2). In individuals with low baseline levels of the respective other bacterial targets, we detected minor effects for *F. prausnitzii* (*p* = 0.03) and borderline effects for *A. muciniphila* (*p* = 0.09) at 25 g RMD (Table 2). While these effects were borderline statistically significant, they did not reach a 2-fold threshold.

## 4. Discussion

We previously reported that the consumption of 25 g/day of RMD increased total fecal bacterial output and number of bifidobacteria but decreased the Firmicutes to Bacteroidetes ratio [2]. Here, we provide further support to the previous observation that adding up to 25 g/day of RMD to the diet is a well-tolerated approach for modifying fecal microbiota towards a potentially more beneficial composition.

Specifically, we now show that dietary supplementation with both 15 g and 25 g/day of RMD increased the amount of fecal *F. saccharivorans* by more than two-fold regardless of the baseline levels (*p* < 0.05). This observation is of interest as such a consistent and significant effect across dietary intake levels is rare when targeting specific fecal bacterial taxa in an intervention study. This finding suggests that RMD, even at a lower intake, can increase the amount of *F. saccharivorans*, which is of interest due to potential associated health benefits. Previous observations were not as strong and occurred at higher intake levels (up to 50 g/day RMD) [13]. A recent study of microbiota differences in patients with UC has shown significantly lower numbers of *F. saccharivorans* compared to healthy controls [8]. It has been suggested that an increase in *F. saccharivorans* may reduce intestinal inflammation as it stimulates the production of anti-inflammatory interleukin-10 in intestinal lamina propria cells isolated from patients with UC [7]. Additionally, in a study where patients with UC received fecal microbiota transplantation, higher *F. saccharivorans* counts in donors were significantly correlated with clinical remission of UC [9].

We also obtained evidence that, in individuals with low baseline levels of *A. muciniphila* and *F. prausnitzii*, RMD supplementation may have increased said taxa compared to the baseline albeit mostly at the higher intake level of 25 g/day. In contrast, we did not detect an effect of RMD in individuals with high baseline levels of the respective bacterial targets. We targeted these species due to their potential to exhibit beneficial effects on the host [4,14]. LAB are bacteria that produce lactic acid as a major end product of carbohydrate fermentation and are frequently used in probiotic formulations due to their beneficial effects (strains from genera *Lactobacillus* and *Bifidobacterium* are most commonly used). While in vitro and rodent in vivo evidence demonstrates anti-inflammatory and immunomodulatory potential of certain LAB species, human trials result in inconsistent findings regarding the association of LAB species with metabolic diseases [5]. *A. muciniphila* is a commensal bacterium for which a low abundance in the gut is recognized as a signature of obesity-related metabolic disorders [14]. As a mucin degrader, *A. muciniphila* is necessary for maintaining optimal thickness of the mucosal layer and the integrity of the intestinal epithelial cells [15], which are components of intestinal health. In rodent studies, prebiotic intake increased *A. muciniphila* abundance in obese and diabetic mouse models [16], while *A. muciniphila* administration reduced endotoxin concentrations in obese mice fed a high-fat diet [17]. In a human trial, supplementation with *A. muciniphila* improved several metabolic parameters related to insulin and lipid metabolism in overweight and obese insulin-resistant participants [4]. In a recent systematic review and meta-analysis of 16 studies, butyrate producer *F. prausnitzii* has been shown to be present in lower abundance in patients with inflammatory bowel disease (IBD) compared to healthy controls, as well as in patients with active IBD compared to patients with IBD in remission [6]. Both *A. muciniphila* and *F. prausnitzii* have been extensively researched in human dietary interventions. Supplementation with ingredients such as inulin-type fructans, fructooligosacharides, polydextrose and soluble corn fiber can increase these species; however, the findings in inulin-oligofructose, resistant starch and wheat bran foods are inconsistent [3]. This inconsistency likely is at least partially due to studies being performed in populations with underlying differences in dietary habits, gut microbiota, health status, etc.

The observation of increases in potentially beneficial bacteria in participants with low baseline levels needs to be interpreted carefully. It might make biological sense that individuals with low baseline levels are more likely to benefit from dietary supplements that can increase fecal numbers of targeted bacteria. However, one also needs to consider that over time, counts of bacteria tend to vary around a mean. Thus, in individuals with a single low baseline measure, the expected trend would be an increase towards the mean, which is what we observed for LAB (see Figure 2). Similarly, in individuals with a high baseline count, the trend in the absence of any intervention effect would be a decrease towards the mean. While observations of changes during the placebo period in comparison to intervention periods can help address this concern, potential remaining carryover effects of intervention periods might have interfered in this analysis. We also note that “high” and “low” baseline levels were set arbitrarily visually based on the observed distribution. However, to demonstrate a meaningful health benefit, cutoffs in future studies should be based on biologically relevant levels that are associated with the likelihood of an increase in a particular microbial taxon rather than statistically determined cutoffs. Further studies that link microbiota composition with well-defined health endpoints are required to establish such biologically relevant levels for each microbial taxon of interest. This issue is further complicated as multiple taxa might mediate similar health benefits and interactions between taxa might also contribute.

As our study provides strong evidence that RMD, even at lower intake levels, increases *F. saccharivorans* regardless of baseline levels, it seems reasonable to explore if RMD can reduce some UC-associated symptoms in future studies. Prebiotics have largely been studied in healthy individuals, often with rather diffuse endpoints that are rarely objectively quantifiable. Various barriers, often regulatory in nature, exist for developing probiotics and prebiotics to target specific disease symptoms, rather than supporting more general and often ambiguous health maintenance claims. It might be timely to invest additional resources into more effectively utilizing the large potential of interventions targeting specific microbiota, including RMD supplements, to improve distinct disease outcomes, especially those associated with the GI tract.

## 5. Conclusions

This secondary analysis of a previously published clinical trial demonstrated an objective, quantifiable effect of RMD on a specific microbial taxon that is potentially beneficial to human health, *F. saccharivorans.* Studies assessing the effects of dietary fibers such as RMD on specific microbial taxa and their related health benefits continue to be warranted.

## Figures and Tables

**Table 1:** qPCR-detected changes in log_10_ counts of targeted fecal bacteria.

Target	Baseline	Placebo	15 g RMD	25 g RMD
*F. saccharivorans*	3.44 × 10^5^	3.35 × 10^5^	6.98 × 10^5^	6.97 × 10^5^
*A. muciniphila*	2.40 × 10^4^	2.35 × 10^4^	3.57 × 10^4^	1.79 × 10^4^
*F. prausnitzii*	2.56 × 10^5^	2.43 × 10^5^	3.14 × 10^5^	2.80 × 10^5^
LAB	2.13 × 10^4^	1.48 × 10^4^	2.43 × 10^4^	1.59 × 10^4^

Mean log_10_ counts of targeted bacterial taxa by qPCR during the intervention trial. *F. saccharivorans*, *Fusicatenibacter saccharivorans*; *F. prausnitzii*, *Faecalibacterium prausnitzii*; *A. muciniphila*, *Akkermansia muciniphila*; LAB, lactic acid bacteria (*n* = 49). Values in bold are significantly different (*p* < 0.05) from both baseline and end of placebo period (0 g).

**Table 2:** *p*-values for exploratory analysis of effects of RMD on *A. muciniphila*, *F. prausnitzii* and LAB in individuals with low baseline log_10_ counts of respective targets.

Target	15 g RMD	25 g RMD
*A. muciniphila*	>0.1	0.09
*F. prausnitzii*	>0.1	0.03
LAB	>0.1	>0.1

*p*-values for differences between baseline and end of intervention in individuals with low baseline log_10_ counts of targeted bacterial taxa by qPCR. *F. prausnitzii*, *Faecalibacterium prausnitzii*; *A. muciniphila*, *Akkermansia muciniphila*; LAB, lactic acid bacteria (*n* = 49).

## Data Availability

De-identified qPCR data will be shared upon request.

## Abbreviations

RMD	Resistant maltodextrin
DF	Dietary fiber
BSS	Bristol stool form scale
GI	Gastrointestinal
UC	Ulcerative colitis
OUT	Operational taxonomic units
WW	Wet weight
LAB	Lactic acid bacteria
*F. saccharivorans*	Fusicatenibacter saccharivorans
*F. prausnitzii*	Faecalibacterium prausnitzii
*A. muciniphila*	Akkermansia muciniphila
